# Perianal Paget's Disease: The 17-Year-Experience of a Single Institution in Taiwan

**DOI:** 10.1155/2019/2603279

**Published:** 2019-10-17

**Authors:** Yu-Chen Wang, Anna Fen-Yau Li, Shung-Haur Yang, Hsiu-Hsun Ma, Wen-Yih Liang

**Affiliations:** ^1^Department of Pathology, Show Chwan Memorial Hospital, Changhua 500, Taiwan; ^2^Department of Pathology and Laboratory Medicine, Taipei Veterans General Hospital, Taipei 112, Taiwan; ^3^School of Medicine, National Yang-Ming University, Taipei 112, Taiwan; ^4^Division of Colon and Rectal Surgery, Department of Surgery, Taipei Veterans General Hospital, Taipei 112, Taiwan

## Abstract

**Aim:**

To determine the incidence, prognosis, and immunophenotypes (CK7, CK20, CDX2, and GCDFP-15) of primary or secondary perianal Paget's diseases (PPDs).

**Methods:**

Twenty-three PPD patients were recruited, including 10 primary and 13 secondary PPDs. Immunophenotypes of PPD were analyzed.

**Results:**

In 23 PPD patients, 14 (60.9%) were male and the median age was 75 years. Three (13.0%, 2 primary and 1 secondary PPDs) had recurrence and two (8.7%, both primary PPDs) had invasive PPDs. The colorectal cancers (CRCs) in secondary PPD cases were located in anorectal area for 9 patients while 4 were located in the rectum; 5, 2, 4, and 2 were in stages I, II, III, and in uncertain stage, respectively. The distant metastasis rates of CRC in the secondary PPD patients during follow-up were 40% (2/5), 0% (0/2), and 50% (2/4) for stages I, II, and III, respectively. Other synchronous or metachronous malignancies included cholangiocarcinoma, urothelial carcinoma, anorectal small-cell carcinoma, and unknown hepatic malignancy. One primary PPD patient died from the metastases of invasive Paget's disease while 3 secondary PPD patients died from the metastases of CRCs during follow-up. Immunohistochemical staining showed CK7 (7/10 and 6/13), CK20 (6/10 and 10/13), CDX2 (6/10 and 12/13), and GCDFP-15 (3/10 and 0/13) positivities in primary and secondary PPD patients, respectively. The immunophenotypes were not statistical significantly related to synchronous CRC (*P* = 0.402, 0.650, 0.127, and 0.068 for CK7, CK20, CDX2, and GCDFP-15, respectively).

**Conclusions:**

The incidence of concurrent CRC in PPD patients is not low. An adequate survey for CRC should be considered for PPD patients at initial diagnosis. In this series of study, stage I CRC with PPD would have a higher metastatic rate, thus indicating aggressive treatment and follow-up. The CK7, CK20, CDX2, and GCDFP-15 immunostaining results for the PPD patients were not predictive of primary or secondary type.

## 1. Introduction

Paget's disease was first described in the breast cancer patients by Sir James Paget in 1874 and was subsequently named after him [1]. It is characterized by the presence of malignant glandular epithelial cells (Paget's cells) within the squamous epithelium. Paget's cells are intraepithelial, large pale cells that contain intracytoplasmic mucinous vacuoles. Paget's disease is relatively rare; it mainly occurs on the nipple and areola (mammary Paget's disease) and infrequently on the vulva, perianal areas, perineal areas, scrotum, and penis (extramammary Paget's disease, EMPD). The origins of the neoplastic cells are presumably hair follicles, sweat glands, and sebaceous glands [1, 2]. Perianal Paget's disease (PPD) was first described by Darier in 1893 [3], 19 years after the first mammary Paget's disease was reported. The incidence of PPD is difficult to estimate accurately due to its rarity; however, it is thought to occur in less than 1-6.5% of all Paget's disease cases [4]. The perianal region accounts for approximately 4.3% of EMPD occurrences and is the second most common location after the vulva [5, 6]. EMPD can be classified as primary or secondary forms sharing similar histology, and the former originates from cutaneous origin and the latter was from anorectal or urogenital carcinomas with intraepithelial spreading [7, 8]. Thus, PPD can also occur either without (primary PPD) or with (secondary PPD) colorectal cancer (CRC) [9–11]. In patients with secondary PPD, skin manifestations would be the initial symptoms the same as the primary PPD cases, such as erythematous change, itching, burning, or pain. We wonder if it is possible to predict occult malignancy in the newly diagnosed PPD patients according to the skin specimen alone before any other clinical survey.

Recently, Kang et al. [12] documented that the activation of the RAS/RAF and PI3K/AKT pathways may have an important role in the pathogenesis of EMPD. However, the cost of genetic testing is relatively high and thus is not practical for general laboratories. Immunohistochemical screening is more convenient and cost-effective for most laboratories. In current concepts, the primary EMPD immunophenotype usually shows cytokeratin 7 (CK7)+/ cytokeratin 20 (CK20)-/ gross cystic disease fluid protein-15 (GCDFP-15)+ while the secondary EMPD shows CK7+/CK20+/GCDFP-15- [13–15]. However, there were some primary EMPD cases showing CK7+/CK20+/GCDFP-15- immunophenotype [13–15], and the different immunophenotypes between primary and secondary EMPD cases may not be so clear-cut. The immunophenotypes of PPD, including CK7 and CK20, have been described [14, 16], with one case of PPD with CDX2 immunoreactivity having been reported [17]. However, no PPD case series have been published on CDX2 expression; and little is known regarding the practical application of CDX2 immunohistochemistry for primary and secondary PPD cases. This study was designed to evaluate the immunophenotypes and long-term prognosis of primary and secondary PPD cases based on our 17-year experience in a single tertiary center in Taiwan.

## 2. Methods

### 2.1. Case Selection and Pathological Review

The institutional review board of Taipei Veterans General Hospital approved the retrospective use of patients' data with a waiver of informed consent (VGHIRB no. 2015-06-005 BC). A retrospective search of surgical pathology database and medical records from January 2000 to December 2016 was performed for patients with PPD treated at Taipei Veterans General Hospital. The term “perianal area” was defined as the “perineal anal triangle” [14]; and vulva and scrotal PPDs were not included. Only histologically proven PPD cases were selected; small specimens that were unsuitable following immunohistochemical studies were excluded. Clinical information, including age, gender, synchronous or metachronous malignancies, recurrent PPD interval, the initial TNM stage of colon cancer, and the metastatic status during follow-up, were obtained from the medical records. All the patients underwent a colonoscopy at the initial PPD diagnosis, and any suspected colorectal malignancies were also confirmed by histology. If a PPD was found with a continuous lesion with CRC or not with a continuous lesion but with a synchronous CRC, it was regarded as “secondary PPD.” Otherwise, it was a “primary PPD.” All the slides were reviewed by two gastroenterology specialized pathologists (W-Y. L. and A. F.-Y. L.).

### 2.2. Immunohistochemistry

Immunohistochemical staining for CK7, CK20, CDX2, and GCDFP-15 was performed for all the included patients. 2 *μ*m thick paraffin sections of the perianal tissue in each case were immunostained using the Leica Bond-Max autostainer (Leica Microsystems GmbH, Wetzlar, Germany). The automated program for immunohistochemistry included deparaffinization using Bond Dewax Solution. Subsequently, antigen retrieval was performed with a citrate solution (pH 6.0) at 100°C for 30 min, prior to incubation with 1 : 100 diluted CK7 antibody (Leica, Newcastle Upon Tyne, UK) at 25°C for 15 min. Alternatively, antigen retrieval was performed with Bond ER2 solution [ethylenediamine tetraacetic acid (EDTA), pH 9.0] at 100°C for 30 min, followed by incubation with 1 : 400 diluted CK20 antibody (Leica, Newcastle Upon Tyne, UK) at 25°C for 15 min. Alternatively, antigen retrieval was performed with EDTA (pH 9.0) at 100°C for 20 min, followed by incubation with 1 : 800 diluted CDX2 antibody (Thermo, Fremont, CA, USA) at 25°C for 15 min. Alternatively, antigen retrieval was performed with EDTA (pH 9.0) at 100°C for 20 min, followed by incubation with 1 : 150 diluted GCDFP-15 antibody (Leica, Newcastle Upon Tyne, UK) at 25°C for 30 min. Visualization was performed using the Bond Polymer Refine Detection kit. Focal positive staining was defined as an expression that was >10% above the negative control in the presence of ≤50% tumor cells. The results of the immunostaining were evaluated by two pathologists (Y.-C.W. and W.-Y.L.).

### 2.3. Statistical Analysis

The association between the immunohistochemical staining results and the primary or secondary PPD was compared using two-sided Fisher's exact test. Focal positive results accounted for positive results in the statistical analysis. IBM SPSS Statistics for Windows, version 24.0 (IBM Corp., Armonk, NY, USA) was used for the statistical analyses. *P* value < 0.05 was considered statistically significant.

## 3. Results

Twenty-four patients with PPD as confirmed by tissue analysis at the Taipei Veterans General Hospital from 2000 to 2016 were included. However, one patient was excluded because the patient's specimen was too small for immunohistochemical analysis, leaving 14 men (60.9%) and 9 women (39.1%) whose ages ranged from 50 to 88 years (mean: 73.7 years; median: 75 years). There were 10 primary PPD cases and 13 secondary PPD cases. The patient demographics in the two groups are summarized in Tables 1 and 2, respectively. Among the 10 primary PPD patients, 2 patients (20.0%, patients 1 and 9) had invasive PPD while the other 8 had noninvasive PPD. Other malignancies were also found in primary PPD cases, including 1 case of cholangiocarcinoma (metachronous, 11 years from initial PPD diagnosis), 1 case of urothelial carcinoma (metachronous, for 2 years), and 1 case of anorectal small-cell carcinoma (metachronous, for 3 years). In the 13 secondary PPD cases, 10 had continuous lesion with CRC (76.9%, 8 with adenocarcinomas and 2 with mucinous adenocarcinomas) while 3 did not have continuous lesion but with synchronous CRC (23.1%, 1 with adenocarcinomas and 2 with mucinous adenocarcinomas) at the time of PPD diagnosis. In addition, there was 1 secondary PPD case with unknown hepatic malignancy (synchronous, without a histological diagnosis, favoring a primary hepatic tumor clinically and radiologically; this patient was also with a synchronous anorectal adenocarcinoma). The location of continuous or synchronous CRC, histologic subtype, initial TNM stage, and interval of distant metastasis occurrence in secondary PPD cases are summarized in Table 3. Nine of the CRCs were located in anorectal areas and 4 were in the rectum; 5 cases were stage I, 2 were stage II, 4 were stage III, and the stages of 2 cases were uncertain due to the loss of follow-up or patient refusal of further evaluation and treatment. Among the 13 secondary PPD patients, 4 patients (30.8%) had distant CRC metastases during the follow-up. Distant metastases were also found in 2 out of the 5 patients with stage I CRC (2/5, 40%), in none of the patients with stage II CRC (0/2, 0%), and in 2 of the 4 patients with stage III CRC (2/4, 50%).

**Figure 1:**
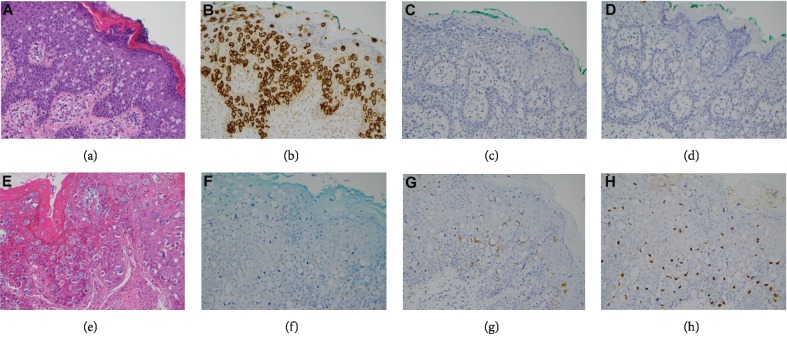
Histology and immunophenotypes of perianal Paget's diseases. (a–d) Patient 8 ((a): H&E; (b): CK7+; (c): CK20-; (d): CDX2-). (e–h) Patient 2 ((e): H&E; (f): CK7-; (g): CK20+; (h): CDX2+) (all photographs presented in 200x magnification).

The morphology and immunophenotypes of primary and secondary PPDs are also documented in Tables 1 and 2; the representative histology of PPD and patterns of immunohistochemical staining are presented in Figure 1. Among the primary PPD cases, there were 7/10 cases (70.0%) with CK7, 6/10 cases (60.0%) with CK20, 6/10 cases (60.0%) with CDX2, and 3/10 cases (30.0%) with GCDFP-15 immunoreactivities, respectively. On the other hand, there were 6/13 (46.2%), 10/13 (76.9%), 12/13 (92.3%), and 0/13 (0.0%) with CK7-positive, CK20-positive, CDX-2-positive, and GCDFP-15-positive in the secondary PPD cases, respectively. The results of the immunohistochemical analysis and the presence of synchronous CRC were analyzed and are summarized in Table 4. The application of CK7, CK20, CDX2, and GCDFP-15 immunohistochemical stains for differentiating immunophenotypes between primary and secondary PPDs was not statistically significant in our cases (*P* = 0.402, 0.650, 0.127, and 0.068 for CK7, CK20, CDX2, and GCDFP-15, respectively).

## 4. Discussion

Here, we have presented 23 PPD patients including primary and secondary cases encountered in a tertiary care center in Taiwan during a 17-year period. To the best of our knowledge, this is the largest series of PPD in Asia currently and the second largest number compared with other currently published literatures [4, 10, 11, 14, 16, 18, 19]. PPD is a relatively rare disease and is believed to originate from the apocrine glands of the perianal skin [8, 20, 21]; there are less than 200 cases reported in the literature. Generally, the clinical presentation of this condition is not specific and frequent symptoms include erythematous changes on the skin, itchiness, burning, pain, and bleeding [19]. There are limited reports of PPD occurring with metachronous or synchronous CRCs; however, these rare conditions are believed to have a poorer prognosis. Although surgery, radiation therapy, photodynamic therapy, topical limiquimod, conventional chemotherapy, and target therapy were conducted in EMPD treatment, there is still no standard management for advanced EMPD including PPD [22]. The natural course of PPD is unclear, either. Therefore, we have documented our experience with 23 primary or secondary PPD patients and have provided the results of their clinical and pathological characteristics.

In our case series, there were 10 primary PPD cases including 3 cases involving other metachronous malignancies, i.e., cholangiocarcinoma, urothelial carcinoma, and rectal small-cell carcinoma (Table 1) and 13 secondary PPD as continuous lesions or synchronous CRCs (including a case of synchronous hepatic tumor) (Table 2). Therefore, several PPD patients (16/23, 69.6%) had an underlying malignancy at the initial diagnosis or during the follow-up period. Almost all these PPD patients sought medical help due to the skin symptoms including erythematous change on the skin, itchiness, burning, or pain initially. Considering the relatively high likelihood of underlying CRCs in PPD, we strongly suggest that colonoscopy should be performed once PPD is diagnosed.

Notably, among the 13 secondary PPD patients, 4 had distant CRC metastases during the follow-up including 2 patients (patients 16 and 19; 2/4, 50.0%) who had T1 or T2 node-negative (stage I) colorectal adenocarcinoma at the initial diagnosis (Table 3). Although the number of cases is too small to determine a statistically significant difference, the proportion of patients with stage I CRC who experienced distant metastases in our series is notably higher than the previously reported incidence [23]. This finding suggests the need for greater caution when treating patients with PPD who have early-stage CRC. If CRC with PPD have a more aggressive clinical course, more aggressive interventions, such as adjuvant chemotherapy and a closer follow-up would be needed to avoid recurrence and distant metastases even without lymph node metastasis at initial diagnosis. In addition, other synchronous or metachronous malignancies, such as cholangiocarcinoma and urothelial carcinoma, were also observed in our series. A more extensive survey, including computed tomography and positron emission tomography, may also be considered for patients with PPD to screen for other underlying malignancies.

In this study, we also performed immunohistochemical analysis for CK7, CK20, CDX2, and GCDFP-15 in skin tissues obtained from patients with primary or secondary PPD. Nowak et al. [16] described 3 of 5 PPD patients with concurrent rectal adenocarcinoma, of which all 3 patients expressed CK7+/CK20+/GCDFP-15-. The other 2 primary PPD cases showed CK7+/CK20-/GCDFP-15+. Goldblum and Hart [14] similarly documented 11 PPD cases, of which 5 secondary PPD patients with rectal adenocarcinomas expressed CK7+/CK20+/GCDFP-15-. Meanwhile, 2 of the 6 primary PPD cases expressed CK7+/CK20+/GCDFP-15- while the other 4 cases showed CK7+/CK20-/GCDFP-15+. Currently, CDX2 immunoreactivity has been documented in only one case report of PPD by Sisodia et al. [17]. Our study revealed that the immunoreactivities for CK7, CK20, CDX2, and GCDFP-15 in primary and secondary PPD cases were 70.0% vs. 46.2%, 60.0% vs. 76.9%, 60.0% vs. 92.3%, and 30.0% vs. 0.0%, respectively. Neither CK7, CK20, CDX2, nor GCDFP-15 was statistical significantly related to the primary and secondary PPD (*P* = 0.402, 0.650, 0.127, and 0.068, respectively; Table 4). Among the secondary PPD patients, up to 12 patients had CDX2+ (12/13, 92.3%) immunophenotype, while only 1 patient (patient 22; 1/13, 7.7%) had CK7+/CK20-/CDX2-/GCDFP-15- immunophenotype. However, there were still 6 primary PPD patients with CDX2 positivity, and comparing CDX2 immunophenotype in all PPD cases with the occurrence of CRCs was not significant, either. In addition, the GCDFP-15 expression was only observed in the primary PPD cases, but the difference between primary (3/10) and secondary (0/13) PPD cases still does not meet the statistical significance. In our case series, CK7/CK20/CDX2/GCDFP-15 immunophenotype of PPD cases appeared not to accurately differentiate primary and secondary PPD cases well. Based on the immunohistochemical staining and clinical presentation, PPD remains an immunohistochemically heterogeneous and complex entity. Since different PPD immunophenotypes share a high incidence of CRCs, colonoscopic examination is suggested for all patients with PPD.

There were some limitations to our retrospective study. First, it is limited by selection bias like all retrospective studies. Second, the included patient number is still relatively small though it is second most in current published studies. Moreover, we did not perform genetic analysis in this study to figure out the genetic details of these PPDs.

Although the number of cases presented in our series was limited, we believe that this study improved our awareness of the clinical presentation and immunohistochemical staining for PPD. Moreover, these findings serve as the important reminders of the potential association between cancers and a poor prognosis in PPD patients. Continued reporting of cases of PPD is essential for identifying reliable parameters by which risk stratification can be performed, as well as for elucidating the nature of the disease.

## 5. Conclusions

Our results demonstrate that PPD patients exhibit a relatively high incidence of concurrent CRC and other metachronous malignancies, and they require multidisciplinary treatment and long-term follow-up. Tissues obtained from patients with PPD would express CK7, CK20, CDX2, and GCDFP-15 with immunochemical stains; however, this staining was not useful in predicting the PPD being primary or secondary (either as a continuous lesion with CRC or with synchronous CRC). In secondary PPD cases, T1 or T2 node-negative CRCs (stage I) with noninvasive PPD may indicate a potential for CRC metastases. More aggressive surveillance may be needed for these patients, even if the patient's CRC was not initially at an advanced stage.

## Figures and Tables

**Table 1:** Patient demographics, pathology, PPD recurrence, synchronous or metachronous malignancies, survival, and immunophenotypes of primary perianal Paget's disease^a^.

Case	Age (years)	Sex	Surgical treatment	Metachronous malignancy	PPD recurrence	Survival and follow-up	Cause of death	CK7 (7/10)	CK20 (6/10)	CDX2 (6/10)	GCDFP-15 (3/10)
1	69	M	APR	Cholangiocarcinoma (metachronous, 11 years)	None	Death, 11 years	Cholangiocarcinoma	-	+	+	-
2	83	F	WLE	None	None	Alive, 11 years	None	-	+	+	-
3	76	M	WLE	None	None	Death, 2 months	Sepsis	+	-	-	+
4	75	M	WLE	None	Yes, 1 year later	Death, 2 years	Unknown (died after discharge)	-	+	+	-
5	87	F	WLE	None	None	Death, 4 years	Respiratory failure	+	F	+	-
6	77	M	WLE	None	None	Death, 9 years	Respiratory failure	+	-	F	-
7	85	M	WLE	None	None	Death, 7 years	Unknown (died after discharge)	F	+	+	-
8	62	F	WLE	Urothelial carcinoma (metachronous, 2 years)	None	Death, 3 years	Urothelial carcinoma with multiple metastases	+	-	-	-
9	60	F	WLE then APR	None	Yes, 4 years later	Death, 6 years	DOD, PPD with lymph nodes and lung metastases	+	+	-	+
10	69	M	WLE	Rectal small-cell carcinoma (metachronous, 3 years)	None	Death, 3 years	Rectal small-cell carcinoma with lymph node metastases	+	-	-	+

^a^F: focally positive. PPD: perianal Paget's disease; APR: abdominal peritoneal resection; WLE: wide local excision; DOD: died of disease.

**Table 2:** Patient demographics, pathology, PPD recurrence, synchronous or metachronous malignancies, survival, and immunophenotypes of secondary perianal Paget's disease^a^.

Case	Age (years)	Sex	Surgical treatment	Continuous lesion or synchronous malignancy	PPD recurrence	Survival and follow-up	Cause of death	CK7 (6/13)	CK20 (10/13)	CDX2 (12/13)	GCDFP-15 (0/13)
11	72	F	APR	Rectal mucinous adenocarcinoma (synchronous, not continuous)	None	Death, 5 months	Respiratory failure	-	-	+	-
12	71	F	WLE+LAR	Rectal mucinous adenocarcinoma (synchronous, not continuous)	Yes, 10 years later	Alive, 11 years	None	-	+	+	-
13	73	M	APR	Anorectal adenocarcinoma (synchronous, not continuous)	None	Alive, 4 years	None	+	+	+	-
14	77	F	WLE	Anorectal carcinoma (continuous); HCC or cholangiocarcinoma (synchronous)	None	Death, 5 months	Liver tumor (suspected HCC or cholangiocarcinoma) with obstructive jaundice and septic shock	+	+	+	-
15	77	M	APR	Anorectal mucinous carcinoma (continuous)	None	Death, 2 years	Anorectal mucinous carcinoma with bone metastasis	+	+	+	-
16	77	M	APR	Anorectal adenocarcinoma (continuous)	None	Death, 4 years	Anorectal carcinoma with bone and lung metastases	F	+	+	-
17	73	M	APR	Anorectal adenocarcinoma (continuous)	None	Alive, 5 years	None	-	-	+	-
18	71	M	N/A	Anorectal adenocarcinoma (continuous)	Unknown	Unknown, lost follow-up	None	-	+	+	-
19	50	F	APR	Anorectal mucinous carcinoma (continuous)	None	Death, 2 years	Anorectal mucinous carcinoma with abdominal cavity, lung, and cerebellar metastases	+	+	+	-
20	88	M	N/A	Anorectal adenocarcinoma (continuous)	None	Death, 1 year	Congestive heart failure	-	+	+	-
21	81	M	APR	Anorectal adenocarcinoma (continuous)	None	Alive, 3 years	None	-	+	+	-
22	79	M	APR	Anorectal adenocarcinoma (continuous)	Unknown	Unknown, lost follow-up	None	+	-	-	-
23	64	F	WLE	Rectal adenocarcinoma (continuous)	None	Alive, 1 year	None	-	+	+	-

^a^F: focally positive. PPD: perianal Paget's disease; APR: abdominal peritoneal resection; WLE: wide local excision; LAR: low anterior resection; HCC: hepatocellular carcinoma; N/A: not applicable.

**Table 3:** Clinicopathological characteristics of continuous or synchronous CRCs in the secondary PPD cases.

Patient	CRC location	Histological type^b^	TNM stage at initial diagnosis	Interval of CRC distant metastasis during follow-up^a^
11	Rectum	Mucinous adenocarcinoma (synchronous, not continuous)	pT3N0Mx (IIA)	N/A
12	Rectum	Mucinous adenocarcinoma (synchronous, not continuous)	pT3N0Mx (IIA)	N/A
13	Anorectum	Adenocarcinoma (synchronous, not continuous)	pT2N0Mx (I)	N/A
14	Anorectum	Adenocarcinoma	pT1N0Mx (I)	N/A
15	Anorectum	Mucinous adenocarcinoma	pT3N2aMx (IIIB)	21 months
16	Anorectum	Adenocarcinoma	pT1N0Mx (I)	24 months
17	Rectum	Adenocarcinoma	pT1N1aMx (IIIA)	N/A
18	Anorectum	Adenocarcinoma	Unknown^b^	Unknown^c^
19	Anorectum	Adenocarcinoma	pT2N0Mx (I)	20 months
20	Anorectum	Adenocarcinoma	cT2N0Mx (I)	N/A
21	Anorectum	Adenocarcinoma	pT3N1bMx (IIIB)	13 months
22	Anorectum	Adenocarcinoma	pT3N2bMx (IIIC)	N/A
23	Rectum	Adenocarcinoma	Unknown^c^	Unknown^d^

^a^N/A: not applicable, free of distant metastases. ^b^Continuous lesion if no note in brackets. ^c^Loss of follow-up. ^d^Patient refused further survey and treatment.

**Table 4:** Correlation between primary or secondary perianal Paget's diseases and immunophenotypes.

	Primary PPD (*N* = 10)	Secondary PPD (*N* = 13)	*P* value^a^
CK7			
Positive	7 (70.0%)	6 (46.2%)	0.402
Negative	3 (30.0%)	7 (53.8%)	
CK20			
Positive	6 (60.0%)	10 (76.9%)	0.650
Negative	4 (40.0%)	3 (23.1%)	
CDX2			
Positive	6 (60.0%)	12 (92.3%)	0.127
Negative	4 (40.0%)	1 (7.7%)	
GCDFP-15			
Positive	3 (30.0%)	0 (0.0%)	0.068
Negative	7 (70.0%)	13 (100.0%)	

CRC: colorectal cancer. ^a^*P* value was analyzed using the two-sided Fisher exact test.

## Data Availability

The data that support the findings of this study are not publicly available due to restrictions.
